# Matrix stiffness may drive multi-cellular crosstalk via YAP signaling in biliary atresia liver fibrosis: a mechanistic review

**DOI:** 10.3389/fcell.2026.1842681

**Published:** 2026-07-01

**Authors:** Jiwen Cheng

**Affiliations:** Department of Pediatrics, First Affiliated Hospital of Xi’an Jiaotong University, Xi’an, Shaanxi, China

**Keywords:** biliary atresia, cellular crosstalk, hepatic stellate cell, matrix stiffness, mechanotransduction, YAP signaling pathway

## Abstract

**Background:**

Biliary atresia (BA) is a devastating liver disease in infants. It leads to progressive cholangitis and fibrosis. In BA, the liver builds up too much extracellular matrix (ECM) and crosslinks it. That makes the tissue stiffer over time—a physical hallmark of fibrosis.

**Mechanistic Framework:**

Most of the current evidence comes from non-BA models and lab studies. Those studies hint that higher matrix stiffness might act as a mechanical trigger. It could activate YAP signaling, which then organizes pathological crosstalk among hepatocytes, cholangiocytes, and hepatic stellate cells (HSCs). Direct proof from BA tissue is still missing.

**Key Findings:**

This review looks at how matrix stiffness—through YAP-driven mechanotransduction—may drive pro-fibrotic changes in hepatocytes, cholangiocytes, and HSCs. We propose a self-reinforcing, multi-cellular pro-fibrotic network: stiff ECM turns on YAP in hepatocytes (which release inflammatory signals) and cholangiocytes (which secrete pro-fibrotic factors). Those activated HSCs then deposit and crosslink even more ECM. That raises stiffness further and locks YAP activation in all 3 cell types. Right now, this model rests mostly on indirect evidence. It needs BA-specific testing.

**Therapeutic Implications:**

We also touch on new strategies that target this mechanobiological circuit—YAP inhibitors and matrix-softening methods.

**Conclusion:**

By pulling together what we know about ECM mechanics, YAP mechanotransduction, and cell-cell crosstalk, this review points to the matrix stiffness–YAP axis as a likely key driver of BA fibrosis. It also looks like a promising treatment target.

## 1 Introduction

Biliary atresia (BA) is the most frequent cause of obstructive jaundice in newborns. It is defined by progressive bile duct destruction and secondary liver fibrosis, which quickly leads to cirrhosis and liver failure (Tam et al., 2024). Liver fibrosis basically means too much ECM deposition and remodeling. That comes with not only disturbed biochemical signals but also major shifts in the physical properties of the tissue environment. A steady increase in matrix stiffness is a central feature (Nikolic and Morici, 2026). In fact, liver stiffness in BA patients correlates strongly with disease progression and native liver survival (Leung et al., 2023).

The ECM is a dynamic, three-dimensional network that gives structural support and mechanical cues to resident cells. In the fibrotic liver, the ECM’s makeup, architecture, and mechanical traits change a lot. Collagens I, III, and IV, fibronectin, and laminin pile up abnormally, while crosslinking enzymes like lysyl oxidase (LOX) are upregulated, producing a stiffer matrix (Zhang et al., 2024a; Liang et al., 2025; Guo et al., 2023). These compositional shifts may actively push disease forward by changing the mechanical microenvironment (Saad et al., 2025).

Recent advances in mechanobiology have shown that cells can detect and respond to ECM mechanics through mechanotransduction pathways. The Hippo/YAP pathway acts as a central mediator, turning extracellular mechanical cues into transcriptional responses (Nikolic and Morici, 2026). YAP is present in developing biliary epithelial cells and can be found in cholangiocytes of the normal adult liver, where it helps regulate bile duct development and repair (Molina et al., 2022). In BA, activated HSCs are the main ECM producers, while reactive, proliferating cholangiocytes are not just targets of fibrosis but also active players in its progression (Zhu et al., 2026).

This review focuses on the idea that increased matrix stiffness may act as a key mechanical signal that activates YAP signaling in hepatocytes, cholangiocytes, and HSCs, thereby building and maintaining a profibrotic positive feedback network. We go over: (1) abnormal ECM remodeling and the resulting stiffness rise in BA; (2) how YAP/TAZ sense and respond to matrix stiffness; (3) stiffness-dependent changes in hepatocytes, cholangiocytes, and HSCs; (4) the proposed profibrotic crosstalk network involving several cell types; and (5) the therapeutic potential of targeting this mechanobiological circuit. Working out how physical forces turn into harmful gene expression in BA is critical for finding new treatment targets. It should be noted that much of the mechanistic evidence discussed comes from non-BA models (e.g., other liver diseases, cancer, or *in vitro* systems), and BA-specific validation is largely absent. So this review presents a testable working hypothesis rather than a fully proven mechanism.

Review methodology: We systematically searched PubMed and Web of Science for literature up to December 2025, using keywords like “biliary atresia”, “matrix stiffness”, “YAP/TAZ”, “mechanotransduction”, “cholangiocyte”, and “hepatic stellate cell”. We gave priority to original research and high-quality reviews that explain mechanical regulation of liver fibrosis, especially those giving direct or indirect evidence relevant to BA.

## 2 Pathological ECM remodeling and increased matrix stiffness in biliary atresia-associated liver fibrosis

### 2.1 Compositional and structural alterations of the ECM

In BA liver tissue, ECM components start piling up and getting crosslinked early. That provides the direct material basis for higher matrix stiffness. Too much of core matrix proteins—collagens I, III, IV, fibronectin, laminin—forms the rigid scaffold of the fibrotic liver (Zhang et al., 2024b). ECM remodeling is very active in BA. A high BA-ECM score goes hand-in-hand with thick, patchy, irregular ECM deposition, which directly reflects how much abnormal matrix has accumulated and become crosslinked (Liang et al., 2025). Changing the tissue’s mechanical microenvironment may actively drive disease progression (Saad et al., 2025).

Why does not the ECM break down enough? It is partly because of an imbalance between matrix metalloproteinases (MMPs) and their tissue inhibitors (TIMPs). In BA, loss of endotoxin tolerance can activate the TLR4/NF-κB pathway, which raises MMP7 expression (Saad et al., 2025). At the same time, a stiff matrix can increase plasma membrane tension through the β1 integrin/RhoA axis, pushing exocytosis and secretion of TIMP-1 (Lachowski et al., 2022). Too much TIMP-1 shuts down MMP activity, reducing collagen and other ECM degradation. That lets the abnormally deposited matrix stick around, creating a self-amplifying pathological cycle (Han et al., 2025).

In this review, ‘hepatic stellate cells (HSCs)’ are the vitamin-A-storing cells in the space of Disse. ‘Portal fibroblasts’ live in the portal tract. Both can turn into myofibroblasts, but their origins, markers, and responses to injury differ (Iwaisako et al., 2014). Current evidence in BA mainly points to HSCs, so we focus on them. When a study specifically looks at portal fibroblasts, we’ll note that.

Liver sinusoidal capillarization, along with the formation of a basement membrane-like matrix, changes the mechanical environment around hepatocytes and cholangiocytes a lot. Higher matrix stiffness can reorganize the cytoskeleton in liver sinusoidal endothelial cells via the FAK-p38-MK2 pathway, making fenestrae fewer or absent (Zhang et al., 2024a). At the same time, abnormal deposits of type IV collagen and laminin inside the space of Disse fundamentally alter the physical structure of liver sinusoids (Wu et al., 2024). Of note, the ECM of the neonatal extrahepatic bile duct is quite heterogeneous in composition and mechanical properties. It is softer and more unevenly distributed than in adults. That structural immaturity may make the bile duct more vulnerable to disease (Li et al., 2023). In BA and other pathological conditions, the ECM around cholangiocytes undergoes sclerotic remodeling, creating a stiff microenvironment that can directly affect cholangiocyte and hepatocyte function through mechanotransduction signals (Li et al., 2021a).

### 2.2 Biophysical detection and clinical relevance of matrix stiffness

To understand how BA liver fibrosis progresses, we need to measure matrix stiffness accurately. High-resolution methods like atomic force microscopy have shown that the overall ECM stiffness of the neonatal rat extrahepatic bile duct is much lower than in adults. The lowest stiffness is at the bile duct margins. That heterogeneity may weaken the tissue’s mechanical integrity (Li et al., 2023). Clinically, shear wave elastography is widely used to measure liver stiffness in children with BA. These measurements correlate well with histological fibrosis stage (Galina et al., 2021; Queiroz et al., 2026).

In BA patients, liver stiffness measurements go up along with serum levels of endoglin, IL-8, and MMP-7. Those serum biomarkers, together with liver stiffness, reflect the active fibrotic and inflammatory processes in the disease (Leung et al., 2023). Also, liver stiffness correlates negatively with native liver survival (Hukkinen et al., 2020) and is closely linked to portal hypertension complications. So clinical monitoring of liver stiffness can help identify patients at high risk of progression (Yeung et al., 2022; Yokoyama et al., 2024). But we still do not know the exact stiffness thresholds for different stages of BA fibrosis, nor how stiffness varies between periductal and perisinusoidal regions and what that means for cell crosstalk.

A major unresolved question is the spatial heterogeneity of matrix stiffness inside the BA liver. The periportal region (where cholangiocytes live) and the perisinusoidal space (where HSCs are) likely have very different mechanical environments. Future studies using high-resolution stiffness mapping—like atomic force microscopy on liver sections—are needed to figure out how each zone contributes to the proposed crosstalk.

## 3 The Hippo/YAP signaling pathway: development, regeneration, and disease

The Hippo signaling pathway is an evolutionarily conserved kinase cascade that controls organ size, tissue homeostasis, and regeneration. Its main effectors, Yes-associated protein (YAP) and transcriptional coactivator with PDZ-binding motif (TAZ), are transcriptional coactivators that shuttle between the cytoplasm and nucleus. When the Hippo pathway is active, a kinase cascade (MST1/2 and LATS1/2) phosphorylates YAP/TAZ, causing their cytoplasmic retention and degradation. When the pathway is off, YAP/TAZ are dephosphorylated and translocate to the nucleus, where they bind TEAD family transcription factors to drive genes involved in proliferation, survival, and migration (Heng et al., 2021).

YAP/TAZ are key regulators of contact inhibition and organ size; their activity is tightly controlled in space and time (Riley et al., 2022). They play essential roles in tissue development, homeostasis, and post-injury regeneration (Meng et al., 2022). Beyond being core effectors of the Hippo pathway, YAP/TAZ also act as hubs that integrate multiple signals from the cellular microenvironment—including growth factors, cytokines, and crosstalk from other pathways (Luo and Li, 2022; Tang et al., 2022)—thereby determining whether cells proliferate, differentiate, survive, or migrate (Harvey and Tang, 2025).

In the liver, YAP has critical roles in bile duct development, controlling cholangiocyte proliferation and ductal plate remodeling (Molina et al., 2022). During liver regeneration after partial hepatectomy, YAP activation is required for hepatocyte proliferation. However, sustained YAP activation contributes to hepatocarcinogenesis and fibrosis. Understanding this context-dependent behavior is essential for interpreting YAP’s role in BA. This section provides the necessary background before we discuss YAP’s mechanotransduction functions below.

## 4 YAP/TAZ as central mechanotransducers of matrix stiffness

### 4.1 Mechanotransduction of matrix stiffness: a signal integration model

**FIGURE 1:**
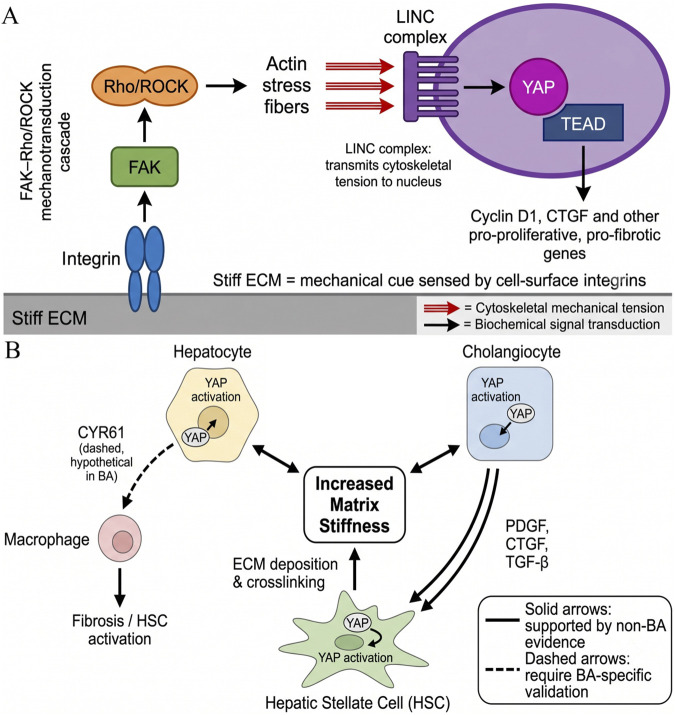
YAP mechanotransduction and the multi-cellular profibrotic network. **(A)** Mechanotransduction of matrix stiffness. Stiff ECM is sensed by integrins, activating the FAK–Rho/ROCK pathway and promoting actin stress fiber formation. Cytoskeletal tension is transmitted to the nucleus via the LINC complex, facilitating YAP nuclear translocation. Nuclear YAP binds TEAD to drive pro-proliferative and pro-fibrotic gene expression. **(B)** Matrix stiffness–YAP axis establishes a multi-cellular profibrotic network. Increased matrix stiffness activates YAP in hepatocytes, cholangiocytes, and HSCs. Activated hepatocytes secrete CYR61 (dashed arrow, hypothetical in BA), recruiting macrophages and promoting inflammation. Activated cholangiocytes secrete PDGF, CTGF, and TGF-β (solid arrows, supported by non-BA evidence), which activate HSCs. Activated HSCs deposit stiff ECM, further increasing matrix stiffness and reinforcing YAP activation in all 3 cell types. Solid arrows: pathways with experimental support in non-BA models; dashed arrows: pathways requiring BA-specific validation.

As introduced in Section 3, YAP/TAZ serve as central mechanotransducers. Cells link ECM mechanics to nuclear gene programs via a multi-level network (Figure 1A), in which YAP/TAZ integrate mechanical signals through the following steps.

(1) Stiff ECM engages integrins, causing them to cluster and recruit focal adhesion complexes. (2) Focal adhesion kinase (FAK) gets activated, turning on downstream Rho/ROCK signaling. (3) Rho/ROCK activation promotes actin stress fiber formation and raises cellular contractility. (4) Cytoskeletal tension is passed to the nucleus through the LINC complex. (5) This mechanical signal helps YAP move into the nucleus, where it binds TEAD transcription factors to turn on pro-proliferative and pro-fibrotic genes. Each of these steps has been validated in non-BA systems, but their relative importance in BA cholangiocytes versus hepatocytes versus HSCs remains to be defined.

#### 4.1.1 Cytoskeletal tension as the central hub

The polymerization state and tension of the actin cytoskeleton—especially F-actin stress fibers—are a key link between outside mechanical stimuli and YAP/TAZ activity. Sticking to a stiff matrix promotes actin stress fiber formation and boosts cellular contractility. This rise in cytoskeletal tension is a major upstream event that activates YAP/TAZ signaling (Virdi and Pethe, 2021; Cai et al., 2021).

#### 4.1.2 Integrin-FAK-Rho/ROCK axis

Integrins are the main sensors of ECM. When they engage with a stiff matrix, they cluster and recruit focal adhesion complexes. Focal adhesion kinase (FAK) is a key player here. Blocking FAK cuts down YAP/TAZ activity (Zarka et al., 2021). Activated FAK triggers downstream signaling cascades, including the Rho/ROCK pathway, which promotes actin stress fiber assembly and further increases cellular contractility (Peng et al., 2025). This Rho/ROCK-mediated cytoskeletal reinforcement is essential for YAP to move into the nucleus in response to matrix stiffness (Nguyen et al., 2021). YAP mechanoactivation happens independently of the canonical Hippo kinase cascade. It involves direct tension-dependent changes in AMOTs (angiomotins), KIBRA, or the actin-capping machinery, which releases YAP from cytoplasmic retention.

#### 4.1.3 The LINC complex and nuclear mechanotransduction

The Linker of Nucleoskeleton and Cytoskeleton (LINC) complex bridges the cytoskeleton and the nuclear lamina. It directly transmits mechanical tension from the cytoplasm to the nucleus. This physical connection helps import mechanosensitive transcriptional regulators like YAP (Takata and Matsumura, 2022). Disrupting the LINC complex impairs YAP nuclear translocation in response to matrix stiffness, highlighting its key role in mechanotransduction.

All together, these multi-level signaling modules work in concert to effectively pass ECM stiffness information through cytoskeletal tension to YAP/TAZ. That makes them core transcriptional coactivators for how cells respond to the mechanical properties of their environment.

### 4.2 Matrix stiffness regulates profibrotic phenotypic changes in hepatocytes, cholangiocytes and hepatic stellate cells via YAP activation

#### 4.2.1 Stiffness-induced hepatocyte YAP activation and inflammatory signaling

Hepatocytes react first to bile acids and mechanical stress in cholestatic liver disease (Cai et al., 2017; Cai and Boyer, 2021). They are not just innocent bystanders. Recent work shows they actively kick off inflammation and fibrogenic signals. How? Accumulated bile acids and higher matrix stiffness switch on YAP inside hepatocytes. That YAP then boosts CYR61 (cysteine-rich angiogenic inducer 61), a molecule that pulls in macrophages. Direct evidence from a NASH mouse model shows that hepatocyte CYR61 secretion promotes pro-inflammatory macrophage polarization and liver fibrosis (Mooring et al., 2023). This mechanism is supported by recent reviews on YAP/TAZ in fibrotic diseases (Mia and Singh, 2022; Lee et al., 2024). Those macrophages release TGF-β and other cytokines, which activate HSCs and drive fibrosis. Knock out YAP in mouse hepatocytes, and fibrosis gets milder—strong evidence that hepatocyte YAP really matters. Does the same happen in BA? And how does it interact with cholangiocyte YAP? We do not know yet; direct tests are needed. Still, adding hepatocytes turns the old linear cholangiocyte–HSC model into a multi-input network.

#### 4.2.2 Stiffness-induced cholangiocyte activation, proliferation, and secretory reprogramming

High matrix stiffness is a hallmark of the liver microenvironment in cholangiopathies like BA. It is also a core biophysical factor that controls cholangiocyte shape and function. When you grow cholangiocytes on a matrix that mimics the stiffness of diseased tissue, they change a lot. YAP moves from the cytoplasm to the nucleus when they sense higher ECM stiffness, which then activates downstream target gene transcription. We’ve seen this in various cell types responding to mechanical cues (Zarka et al., 2021; Peng et al., 2025). As described in Section 4.1, mechanical signals from stiff ECM go through integrin-FAK-Rho/ROCK and the LINC complex, eventually pushing YAP into the nucleus (Zarka et al., 2021; Peng et al., 2025; Nguyen et al., 2021; Takata and Matsumura, 2022). This may drive upregulation of cell cycle genes like Cyclin D1 and c-Myc, leading to abnormally active cholangiocyte proliferation. Histologically, that shows up as a markedly increased and expanded ductular reaction (Hukkinen et al., 2020).

In BA and similar cholangiopathies, injured cholangiocytes change a lot. They start making a completely different set of secreted factors. Studies confirm that activated cholangiocytes pour out PDGF, CTGF, TGF-β, and IL-6 (Jalan-Sakrikar et al., 2025). CTGF is a classic Hippo-YAP target gene. When CTGF goes up, it tells you cholangiocytes are activated—and it strongly pushes collagen deposition (Xie et al., 2024). All these secreted factors together form a complex paracrine web. That web directly hits HSCs and portal fibroblasts, making them activate, multiply, and crank out even more ECM (Wang et al., 2025).

YAP activation may also help cholangiocytes resist cellular senescence caused by cholestatic and inflammatory stress. In the BA pathological environment, sustained YAP signaling might allow cholangiocytes to escape senescence by suppressing p53-related pathways or upregulating anti-apoptotic and pro-survival genes. Right now, we do not know whether YAP has pro-fibrotic versus pro-repair functions in cholangiocytes depending on the stage.

#### 4.2.3 Controversial role of cholangiocyte epithelial-mesenchymal transition (EMT)

Could cholangiocytes undergo EMT and contribute to BA liver fibrosis? Some studies suggest that’s possible. They’ve found a lot of EMT and fibrosis-related pathway activity in BA cholangiocytes, with lower epithelial markers (like E-cadherin) and higher mesenchymal markers (like vimentin and N-cadherin) (Meng et al., 2026). Direct evidence in BA is missing, but studies in other liver disease models hint that mechanical stress can cause ROS production and YAP activation, which together might promote EMT (Du et al., 2025).

Functional experiments show that lowering the m6A methyltransferase METTL3 in BA cholangiocytes boosts their migration and pushes EMT forward (Meng et al., 2026). Also, overexpressing SULT2B1 in BA liver tissues promotes cholangiocyte EMT via the Wnt/β-catenin/MMP7 pathway. Its expression level correlates strongly with fibrosis severity and poor outcome (Yang et al., 2025). Recent single-cell transcriptomic studies in BA livers suggest some cholangiocytes co-express epithelial and mesenchymal markers, hinting at a partial EMT state. But we still do not know how much these cells add to the myofibroblast pool (Xiao et al., 2025).

But we have to be careful: the functional importance of EMT in liver fibrosis is hotly debated. Several lineage-tracing studies have questioned whether EMT-derived fibroblasts really contribute much to the myofibroblast pool in liver fibrosis (Chu et al., 2011; Munker et al., 2017). Given this controversy, here’s what we think: the functional contribution of cholangiocyte EMT to matrix deposition in BA is not proven. The molecular changes people see might just reflect cellular plasticity—cells changing their shape and markers—not a real conversion into matrix-producing myofibroblasts. What we badly need are lineage-tracing studies in BA-relevant models.

#### 4.2.4 Stiffness-dependent activation and phenotypic maintenance of hepatic stellate cells

In a normal liver, quiescent HSCs live in the soft perisinusoidal space of Disse. During fibrosis, too much ECM deposition makes matrix stiffness rise sharply (Zhao et al., 2023). That stiffened ECM gives a critical mechanical cue for HSC activation (Saad et al., 2025). When you grow HSCs on a rigid matrix that mimics the stiffness of fibrotic tissue, they activate and turn into myofibroblasts (Zhao et al., 2023; Chen et al., 2023). As described in Section 4.1, this comes with integrin β1 activation and YAP nuclear entry and activation (Chen et al., 2023). Compared to cells on soft matrices, HSCs on stiff matrices show higher levels of α-SMA and collagen I (Liu et al., 2021; Jia et al., 2026). Seminal work by Mannaerts et al. (2015) first showed that YAP controls HSC activation. Later studies found that ω-3 PUFAs encourage YAP/TAZ degradation, thereby blocking HSC activation (Zhang et al., 2016).

YAP activity is crucial for keeping HSCs activated. HSCs grown on stiff matrices pick up mechanical signals through integrin-based focal adhesions and other mechanoreceptors. That leads to YAP dephosphorylation and nuclear entry (Jia et al., 2026; Zheng et al., 2023). Nuclear YAP then turns on a range of pro-fibrotic genes, driving and sustaining the activated HSC phenotype (Jia et al., 2026). The deubiquitinase OTUD5 plays a central part in this stiffness-induced HSC activation. It removes K48-linked ubiquitin chains from YAP, preventing YAP breakdown (Jia et al., 2026). On the flip side, culturing already-activated HSCs on a low-stiffness matrix causes YAP to be inactivated and leave the nucleus, along with HSC apoptosis or a return toward a quiescent state (Chen et al., 2023). That shows stiffness-dependent HSC activation is dynamic and reversible.

#### 4.2.5 Enhanced ECM production and remodeling by activated hepatic stellate cells

Activated HSCs are the main source of excess ECM during liver fibrosis. They not only make large amounts of structural matrix proteins, but also release various enzymes that control ECM crosslinking and degradation balance. Studies show that activated HSCs have much higher expression of α-SMA and COL1A1 (Rodríguez-Rodríguez et al., 2021). The zinc finger protein ZNF469 goes up during HSC activation and may act as a transcription factor that positively regulates several collagen and proteoglycan genes (Ariyachet et al., 2024).

As a central mechanotransduction mediator, YAP directly regulates the ECM-producing capacity of HSCs at the gene level. When they sense higher matrix stiffness, activated HSCs activate YAP and send it to the nucleus, where it directly starts transcription of ECM structural genes like type I collagen and fibronectin (Aoyama et al., 2023). YAP also controls LOX family members, which are involved in ECM crosslinking. So YAP activation in HSCs promotes both the production of ECM “raw materials” and the “reinforcement” of deposited matrix through crosslinking.

YAP-driven ECM production and crosslinking lead to a further rise in local stiffness. That heightened matrix stiffness then acts as a strong mechanical signal, continually activating and keeping YAP active in HSCs. That builds a self-amplifying positive feedback loop: Stiffness → YAP → ECM production/crosslinking → Increased stiffness (Han et al., 2025; Caon et al., 2024). Intervening in this loop has become a new idea for anti-fibrotic therapy.

## 5 A multi-cellular mechanobiological network: integrating hepatocyte, cholangiocyte, and hepatic stellate cell crosstalk via the matrix stiffness–YAP axis

Crucial Caveat: The model in Section 5 brings together mechanobiological principles from non-BA liver diseases, cancer, and *in vitro* systems. Direct experimental evidence for each step of this crosstalk network within BA livers is currently missing. So this model should be seen as a testable working hypothesis to guide future BA-specific research.

### 5.1 Hepatocyte YAP activation as an upstream initiator of inflammation and fibrosis

As discussed in Section 4.2.1, hepatocyte YAP activation by increased matrix stiffness and bile acids promotes CYR61 secretion, attracts macrophages, and starts inflammation. This inflammatory environment further activates cholangiocytes and HSCs, making hepatocytes a key upstream node in the profibrotic network (Mooring et al., 2023; Mia and Singh, 2022; Lee et al., 2024).

### 5.2 Bidirectional cross-regulation of paracrine and mechanical signals: the cholangiocyte–HSC axis

During BA liver fibrosis, cholangiocytes and HSCs may engage in a continually reinforced, two-way loop driven by both paracrine and mechanical signals. At the heart of this proposed loop is the idea that increased matrix stiffness activates YAP signaling, which may push cholangiocytes to secrete pro-fibrotic factors that then activate nearby HSCs. As shown in Figure 1B, when they sense higher matrix stiffness, cholangiocytes move YAP to the nucleus and activate it (Zhu et al., 2026; Molina et al., 2022). Activated YAP drives the expression and secretion of downstream targets, including CTGF and PDGF.

Direct evidence for such YAP-dependent paracrine signaling between cholangiocytes and HSCs in BA is currently lacking. Although YAP-dependent paracrine signaling has been seen in other fibrotic conditions, whether it happens in BA remains a major gap. We need direct study using BA-specific co-culture systems and conditional knockout models.

Once activated, HSCs turn into myofibroblasts and make and deposit large amounts of stiff ECM parts, mostly collagens. That raises tissue stiffness further. This higher matrix stiffness then acts as a powerful mechanical signal, giving continuous feedback that strengthens YAP activation in both cholangiocytes and HSCs. So an initial injury or inflammation that raises ECM stiffness activates YAP in cholangiocytes, encouraging the release of pro-fibrotic factors. Those factors further activate HSCs, which produce and deposit more rigid ECM. This newly formed rigid ECM serves as an even stronger mechanical stimulus, back-activating YAP in both cell types. That ultimately creates a self-perpetuating, ever-amplifying pathological positive feedback loop. This model remains hypothetical until direct experimental validation in BA tissues appears.

### 5.3 Extracellular vesicles as potential mediators of YAP signal transmission

Inside the fibrotic microenvironment, extracellular vesicles (EVs) act as important messengers between cells. They carry various bioactive molecules that can change YAP signaling activity in recipient cells. We’ve seen similar mechanisms in several tumor models. For example, in gastric cancer, cancer-associated fibroblast-derived EVs rich in annexin A6 activate FAK-YAP signaling in recipient cancer cells (Uchihara et al., 2020). In colorectal cancer, cancer-associated fibroblast-derived small EVs carrying lncRNA WEE2-AS1 block the Hippo pathway by promoting MOB1A ubiquitination and degradation, leading to YAP nuclear entry (Yang et al., 2022).

Whether similar EV-mediated YAP signal transmission happens between cholangiocytes and HSCs in BA has not been studied. It is a promising direction for future work. EVs hold potential as both biomarkers and therapeutic targets for BA-associated liver fibrosis.

## 6 Potential therapeutic avenues: Preclinical evidence and hypothetical frameworks

### 6.1 Strategies targeting YAP/TAZ activity

Targeting the YAP/TAZ pathway might help suppress BA-linked liver fibrosis. Right now, most research is preclinical—animal studies. No clinical trials have started for BA. A key challenge is to get liver-specific targeting so we avoid side effects elsewhere.

#### 6.1.1 Direct inhibition of YAP-TEAD interaction

Verteporfin is a widely studied inhibitor of YAP-TEAD binding. It breaks their interaction and blocks transcription of downstream pro-fibrotic genes (Zhu et al., 2026). In a rhesus rotavirus-induced BA mouse model, verteporfin treatment eased liver fibrosis and improved cholangiocyte permeability and junctional integrity (Zhu et al., 2026). Beyond BA models, verteporfin has also shown benefit in mouse models of polycystic liver disease and glycogen storage disease type Ia. That further supports YAP inhibition in cholestatic and fibrotic liver conditions (Masyuk et al., 2022; Xie et al., 2022). Also, natural products like Chikusetsusaponin IVa (which directly binds YAP) and luteolin have been shown to reduce liver fibrosis by targeting the YAP/TAZ pathway (Gao et al., 2025; Wang and Lan, 2025).

#### 6.1.2 Indirect modulation of YAP/TAZ activity

Statins can indirectly block YAP/TAZ nuclear entry by suppressing Rho GTPase activity (Russell and Camargo, 2022). Activating AMPK can inhibit YAP/TAZ signaling and improve liver fibrosis (Shihan et al., 2024). Vitamin D receptor agonists reduce liver fibrosis by lowering YAP transcriptional activity (Wang et al., 2024). Targeting acid ceramidase has also been shown to block YAP/TAZ signaling and reduce fibrosis in mouse models (Alsamman et al., 2020). Moreover, combining YAP inhibition with BRD4 inhibition has been suggested as a new anti-fibrotic approach (Zhubanchaliyev et al., 2016). Some of these agents are already used clinically for other diseases, giving them a good safety profile and a chance for repurposing in BA.

#### 6.1.3 Gene silencing technologies

Knocking down YAP1 or TAZ expression with siRNA or shRNA has been shown to block the pathway and effectively stop fibrosis progression (Russell and Camargo, 2022). A new bispecific siRNA (bsYW-61) can simultaneously target conserved sequences in the YAP1 and WWTR1 genes, achieving joint knockdown of both key transcriptional coactivators (Ueda et al., 2025). A key optimization direction is to develop liver-targeted delivery systems to get more drug into cholangiocytes and HSCs.

### 6.2 Strategies targeting the matrix mechanical microenvironment

#### 6.2.1 Inhibiting ECM deposition

The anti-fibrotic drug pirfenidone can indirectly affect ECM deposition and stiffness by lowering collagen production and TGF-β signaling (Bai et al., 2025). Its action partly involves the integrin/YAP signaling pathway.

#### 6.2.2 Creating a matrix-softening microenvironment

Matrix-softening hydrogels or injectable mechanically adaptive biomaterials could serve as cell carriers or drug delivery systems to create a local “softening” environment (Guixé-Muntet et al., 2020). Given the immature, low-stiffness nature of the neonatal extrahepatic bile duct ECM (Li et al., 2023), making biomaterials that mimic or encourage a healthy, soft ECM might help disrupt abnormal cell interactions in BA.

#### 6.2.3 Reducing matrix stiffness by inhibiting collagen crosslinking

LOX inhibitors can lower matrix stiffness by reducing collagen fiber crosslinking. They’ve shown efficacy in other liver fibrosis models. LOX levels in BA children correlate with liver stiffness measurements, pointing to a significant role for LOX-mediated collagen crosslinking in matrix stiffening during BA liver fibrosis (Leung et al., 2023).

#### 6.2.4 Blocking upstream mechanotransduction

Targeting key nodes of mechanotransduction pathways—like integrins, FAK, and RhoA/ROCK—can lessen stiffness-induced HSC activation (Sakai and Yoshimura, 2021). FAK inhibition (e.g., PF-562,271) not only blocks integrin-mediated cytoskeletal tension but also directly cuts YAP nuclear enrichment by disrupting the FAK-Src-p130Cas signaling axis (a key mechanotransduction pathway). Targeting upstream signaling molecules could reduce the flow of mechanical signals to YAP, possibly avoiding the systemic side effects that come with direct YAP inhibition.

### 6.3 Stage-specific comprehensive treatment strategies: a hypothetical framework

The stage-specific strategy below is completely hypothetical. It is presented only to spark discussion and future research. It has no current experimental or clinical support in BA.

**FIGURE 2:**
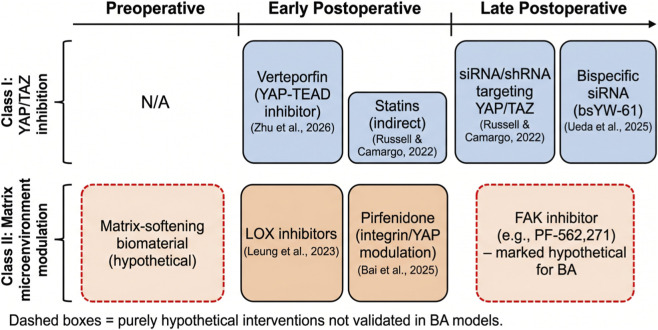
Hypothetical stage-specific therapeutic interventions targeting the matrix stiffness–YAP axis. Interventions are classified into YAP/TAZ inhibition (Class I) and matrix mechanical microenvironment modulation (Class II). A speculative timeline (preoperative, early postoperative, late postoperative) is shown to illustrate how different strategies might be aligned with disease stages. Dashed boxes denote purely hypothetical interventions that have not been validated in BA models. References for each intervention are provided where available (e.g., verteporfin: Zhu et al., 2026; LOX inhibitors; Leung et al., 2023; statins; Russell and Camargo, 2022).

The vicious cycle between matrix stiffness and YAP activation suggests that combining mechanical microenvironment modulation with YAP pathway inhibition might be promising. The hypothetical stage-specific approach sketched in Figure 2—preoperative matrix softening to help surgery, early postoperative YAP inhibition to stop fibrosis from starting, and late postoperative matrix degradation to handle established fibrosis—could in theory match the natural history of BA. All these strategies are still at the conceptual stage and need systematic preclinical testing. Dashed boxes in Figure 2 stand for purely hypothetical interventions.

### 6.4 Current preclinical models for BA: applicability and translational gaps

Moving therapies that target the matrix stiffness–YAP axis forward needs careful testing in BA-specific preclinical models. The rhesus rotavirus (RRV)-induced mouse model is still the most used immunocompetent model. It captures key features of BA, including cholangitis and progressive fibrosis. But we need more study on when and how much matrix stiffening happens in this model. Alternatively, surgical bile duct ligation (BDL) in neonatal mice gives a technically reproducible model of obstructive cholestasis, though it lacks the inflammatory trigger central to BA pathogenesis. Organoid-based systems and stiffness-tunable biomaterial platforms offer promising ways to study cell-autonomous and crosstalk mechanisms under defined mechanical conditions.

So far, verteporfin has shown efficacy in the RRV model (Zhu et al., 2026). But the effects of matrix-softening strategies or YAP-targeting agents delivered with liver-targeting vehicles have not been systematically tested in BA models. Future translational studies should focus on: (1) tracking matrix stiffness changes over time in BA models; (2) directly comparing treatment effects when interventions start at different disease stages; and (3) setting up BA-relevant pharmacokinetic and pharmacodynamic models to test liver-targeted agents. Combining YAP/TAZ inhibition with other epigenetic modulators (e.g., BRD4 inhibitors) as suggested by Zhubanchaliyev et al. (2016) is worth exploring in BA models.

## 7 Current research gaps, controversies, and future perspectives

### 7.1 Key research gaps

#### 7.1.1 Mechanistic gaps

Even though we have growing evidence linking matrix stiffness to YAP activation in BA, several mechanistic questions are still open. First, the YAP mechanotransduction pathways in hepatocytes, cholangiocytes, and HSCs may differ. We do not know if the mechanosensing of immature neonatal bile duct ECM involves unique YAP-regulatory molecules. Second, we need systematic studies on how YAP activation and the classic TGF-β/Smad pathway cooperate or oppose each other during HSC activation. Also, we need to know if YAP activation levels differ at different stiffness thresholds. Third, the regulatory network between ECM crosslinking enzymes and YAP is still poorly understood, especially the specific role of LOX family members in YAP-mediated matrix stiffening. Fourth, we need to figure out the exact molecular points where inflammatory and mechanical signals work together to regulate YAP in BA, and which signaling mode dominates at different disease stages. Fifth, we have not sorted out the relative contribution of hepatocyte YAP versus cholangiocyte YAP to fibrosis initiation and progression in BA. Cell-type-specific knockout models are urgently needed. Sixth, direct spatial evidence—for example, seeing YAP nuclear translocation together with pro-fibrotic factor expression in BA cholangiocytes *in situ*—is completely missing. That’s a critical priority.

#### 7.1.2 Cell specificity and spatiotemporal dynamics gaps

The stage-dependent role of YAP in cholangiocytes—whether it helps repair or drives fibrosis—and its functional weight in the “proliferation-secretion-EMT” axis remain unclear. Also, we need lineage tracing to find out whether cholangiocyte EMT in BA is complete or partial, and how much EMT-derived cells directly add to liver fibrosis. Additionally, we do not know the specific matrix stiffness thresholds at different stages of BA liver fibrosis, nor how spatial stiffness heterogeneity (periductal versus perisinusoidal areas) affects cell crosstalk. A major unresolved question is the spatial heterogeneity of matrix stiffness within the BA liver. The periportal region (where cholangiocytes live) and the perisinusoidal space (where HSCs are) likely have very different mechanical environments. Future studies using high-resolution stiffness mapping—like atomic force microscopy on liver sections—are needed to dissect zone-specific contributions to the proposed crosstalk. The effect of bile acid buildup on cholangiocyte mechanosensing—possibly through cytoskeletal reorganization or oxidative stress-mediated YAP activity regulation—also needs study.

#### 7.1.3 Translational gaps

Could EVs from activated cholangiocytes or HSCs be used as biomarkers in BA? That has not been explored, especially whether these EVs carry specific molecules that regulate YAP activity. Also, we lack BA-specific YAP inhibitor delivery systems with good liver-targeting properties. There’s also no non-invasive diagnostic and prognostic system that combines liver stiffness measurements with YAP pathway-related biomarkers. Finally, we have not defined stage-specific treatment protocols tailored to different clinical courses of BA.

### 7.2 Core controversies

Three core controversies currently shape the field. First, the function of YAP in BA cholangiocytes is still debated. Yes, YAP drives abnormal cholangiocyte proliferation and pro-fibrotic factor secretion. But its physiological role in biliary injury repair is disputed. It seems plausible that YAP has a context-dependent function. It might switch from a pro-repair mediator in the early, acute injury phase to a pro-fibrotic driver under long-lasting mechanical stress and inflammation in the progressive phase. That switch may depend on how strong and long YAP activation lasts, its crosstalk with other pathways like Notch and Wnt, and the state of cholangiocytes (e.g., proliferative vs. senescent). Second, the contribution of cholangiocyte EMT to liver fibrosis is contested. We do not know what share of cholangiocytes undergo EMT in the fibroblast pool, nor how much they directly add to ECM production (see Section 4.2.3). Third, the best timing for matrix stiffness intervention is unknown. We do not know at which specific stage of BA matrix stiffness starts to rise significantly, nor whether intervening early versus late makes a difference. Solving these controversies will require lineage tracing studies, cell-type-specific gene knockout models, and long-term stiffness monitoring in BA patients.

### 7.3 Future perspectives

To better understand the matrix stiffness–YAP axis, we need cell-specific gene knockout models to tease apart the contribution of YAP activity in different cell types. Using cell-specific YAP/TAZ knockout mice could help define how YAP activity in different cell types adds to BA liver fibrosis. For instance, in a cholestatic fibrosis model, knocking out the JCAD gene specifically in HSCs greatly reduced bile duct ligation-induced liver injury and fibrosis (Xie et al., 2024). The role of cholangiocyte-derived YAP activity in responding to changes in the matrix mechanical environment and influencing nearby HSCs still needs to be clarified using cholangiocyte-specific YAP/TAZ knockout models.

Looking at how inflammatory and mechanical signals work together is key to understanding the spatiotemporal dynamics of BA liver fibrosis. YAP/TAZ are central hubs that take in many physical and biochemical signals (Li et al., 2021b). In BA, ongoing inflammation may team up with increased matrix stiffness to boost YAP/TAZ nuclear entry and transcriptional activity, possibly by changing upstream molecules like integrins and FAK. For example, higher ECM stiffness can cause liquid-liquid phase separation of the DDR1 receptor, which blocks the Hippo pathway kinase LATS1 and thus activates YAP (Liu et al., 2023). Also, bile acid buildup may alter how sensitive cells are to matrix stiffness by causing oxidative stress or affecting cytoskeletal organization, thereby changing YAP activity. These mechanisms need more study in the context of BA’s disease timeline.

Future mechanistic studies should also spell out the differences in YAP mechanotransduction pathways between hepatocytes, cholangiocytes, and HSCs in BA. We need to identify cell type-specific upstream sensors, activation thresholds, and downstream target gene profiles. At the same time, exploring how spatial heterogeneity of matrix stiffness shapes cell niches will give a theoretical foundation for precise intervention. Translational challenges—liver-specific targeting and finding non-invasive diagnostic markers—are discussed in Sections 6.1–6.3.

## 8 Discussion

So what have we learned? In this review, we’ve pulled together current evidence to argue that increased matrix stiffness is not just a side effect of BA fibrosis. It may actively drive the disease, mainly through YAP signaling. Our key idea is this: the matrix stiffness–YAP axis creates a self-amplifying, multi-cellular pro-fibrotic network that pulls in hepatocytes, cholangiocytes, and HSCs. That gives United States of America coherent mechanobiological framework—one that links ECM mechanics, cellular mechanotransduction, and cell-cell crosstalk in BA. But we have to be honest: much of this framework rests on indirect evidence from non-BA models. BA-specific validation is badly needed.

Why does this framework help? It explains several puzzling features of BA fibrosis. First, why fibrosis keeps going once it starts: once stiffness rises, the mechanical environment itself becomes a driver, reinforcing its own progression through YAP-dependent cellular responses. Second, it sees hepatocytes and cholangiocytes not as passive victims but as active players—their YAP-dependent secretory changes may start and sustain HSC activation. Third, it puts YAP at the center where mechanical and biochemical signals converge. That fits with why YAP activity tracks with disease severity, and why blocking YAP looks promising in preclinical models.

What about treatment? The matrix stiffness–YAP axis gives us several places to intervene. Direct YAP inhibition, matrix-softening strategies, and blocking upstream mechanotransduction all hit different nodes. Combining them might work even better. The stage-specific idea we proposed—matching interventions to BA’s natural history—recognizes that the disease changes over time. But let’s not get ahead of ourselves. These strategies are mostly preclinical right now. Rigorous testing in BA-specific models is a must before we can think about the clinic.

We also need to admit the limits of current research—and of this review. First, direct evidence for YAP-dependent paracrine signaling between hepatocytes, cholangiocytes and HSCs in BA just is not there yet. We’re mostly guessing from other diseases. Second, we do not know the exact stiffness thresholds that turn on YAP in each cell type. We also do not know how stiffness varies across different regions of the BA liver. Third, whether YAP in cholangiocytes helps repair or drives fibrosis likely depends on disease stage—but that’s still unclear. Lineage tracing and conditional knockouts could help.

Bottom line: this review highlights the matrix stiffness–YAP axis as a possible key driver of BA fibrosis and a promising therapy target. By laying out a detailed molecular framework that connects ECM mechanics to nuclear gene programs in a disease-specific way, we hope to push more research into the mechanobiology of BA—and speed up the development of targeted anti-fibrotic treatments.
